# Investigating the *in vitro* antibacterial, antibiofilm, antioxidant, anticancer and antiviral activities of zinc oxide nanoparticles biofabricated from *Cassia javanica*

**DOI:** 10.1371/journal.pone.0310927

**Published:** 2024-10-01

**Authors:** Mohammed S. Almuhayawi, Mohammed H. Alruhaili, Mohamed K. Y. Soliman, Muyassar K. Tarabulsi, Ruba A. Ashy, Amna A. Saddiq, Samy Selim, Yasir Alruwaili, Salem S. Salem

**Affiliations:** 1 Department of Clinical Microbiology and Immunology, Faculty of Medicine, King Abdulaziz University, Jeddah, Saudi Arabia; 2 Yousef Abdulatif Jameel Scientific Chair of Prophetic Medicine Application, Faculty of Medicine, King Abdulaziz University, Jeddah, Saudi Arabia; 3 Special Infectious Agents Unit, King Fahad Medical Research Center, King AbdulAziz University, Jeddah, Saudi Arabia; 4 Botany and Microbiology Department, Faculty of Science, Al-Azhar University, Nasr City, Cairo, Egypt; 5 Department of Basic Medical Sciences, College of Medicine, University of Jeddah, Jeddah, Saudi Arabia; 6 Department of Biological Sciences, College of Science, University of Jeddah, Jeddah, Saudi Arabia; 7 Department of Clinical Laboratory Sciences, College of Applied Medical Sciences, Jouf University, Sakaka, Saudi Arabia; 8 Sustainable Development Research and Innovation Center, Deanship of Graduate Studies and Scientific Research, Jouf University, Sakaka, Saudi Arabia; Cairo University, Faculty of Science, EGYPT

## Abstract

It is thought to be risk-free, environmentally benign, and safe for biological processes to produce zinc oxide nanoparticles from renewable resources. This study examined *Cassia javanica’s* ability to create ZnONPs. The generated ZnONPs were analyzed using a variety of techniques, such as TEM, FTIR spectroscopy, UV-Vis spectroscopy, and XRD analysis. The antibacterial potential of ZnONPs has been investigated using both Agar well diffusion and microtitreplate (MTP) methods. One method used to evaluate ZnONPs’ capacity to scavenge free radicals at different concentrations was the DPPH method. The permanent zinc oxide (ZnO) shape and the naturally occurring crystal structure of ZnONPs were validated by the XRD data. ZnONPs showed antibacterial activity with MICs of 31.7 μg/mL toward *Bacillus subtilis*, 62.5 μg/mL for *Salmonella typhimurium*, *Escherichia coli* while *Clostridium sporogenes* and *Bacillus pumilus* was 125μg/mL. Furthermore, ZnONPs demonstrated a range of antibiofilm activities toward *Staphylococcus aureus* (MRSA). ZnONPs showed an intriguing antioxidant capacity, achieving IC_50_ of 109.3 μg/ml μg/mL. Additionally, ZnONPs demonstrated low toxic effect on Vero cell with IC_50_ 154.01 μg/mL as well as possible anticancer action when applied to the carcinoma cell lines HepG2 with IC_50_ of 47.48 μg/mL. Furthermore, ZnONPs at 62.5 μg/mL had a promising antiviral impact against HSV1 and COX B4, with antiviral activities of 75.4% and 65.8%, respectively.

## Introduction

Arguably among the most significant and active areas associated with material science study nowadays is nanotechnology. According to distinct parameters including dimensions, variation, and shape, nanoparticles have recently become the subject of completely novel and advanced study [1, 2]. The field of nanomaterials is one that is constantly evolving and has an impact on every aspect of existence for humans [3–7]. A variety of approaches were originally employed to generate NPs, but now environmentally friendly, more secure, more affordable, and less hazardous techniques—achieved through green techniques are being sought for [8–10]. Chemical creation of NPs presents a variety of issues; thus, their application is not advised, particularly in the medical domain. Furthermore, the chemical method needs costly chemicals to generate nanoparticles [11] The other option to chemical-based remedies, which have hazards, is green which are environmentally friendly options that rely on microbes and plants [12, 13]. Because naturally derived sources that involve different plant organ extraction and different microorganisms are used, the green approach to generating NP decreases the usage of dangerous compounds [14–17]. Researchers are becoming interested in using plants for the production of different NPs since they are readily available and pose little potential hazard [18, 19].

ZnO nanoparticles have many positive effects on the ecosystem and are similar to different therapeutic purposes in healthcare. Green compounds are becoming more and more sustainable, precisely calculated in huge numbers, and currently there really is no compelling explanation to employ extreme amounts of power, temperature, or biosynthesis [20]. The implementation of nanotechnologies within our daily lives has made nano pharmaceuticals, which include drug as well as medical nanomaterials, helpful nanomaterials utilized as antimicrobial substances or multifunctional nanostructures employed in the recognition of biomarkers as well as nanochips [21–23]. Numerous studies investigated ZnONPs towards harmful microorganisms that affect humans. ZnONPs’ inhibitory effect on methicillin-resistant *Staphylococcus aureus* [24] and the presence of spontaneous, resistant to multiple drugs *Acinetobacter baumannii* had been noted [11]. Additionally, it has demonstrated antibacterial efficacy versus *Salmonella typhi*, *Shigella fexneri*, *Vibrio cholerae*, *Streptococcus mutans*, and *Streptococcus pyogenes* [25]. Previous investigations have demonstrated the link between ZnONPs diameter and antibacterial activity [26]. ZnO NPs with 8 nm in size shown inhibitory effects on *E*. *coli*, *S*. *aureus* and *B*. *subtilis* although larger sizes had marginal antimicrobial actions [27]. The researchers examined the processes by which Zinc Oxide Nanoparticles inhibited the development of various microorganisms, including *Bacillus subtilis*, *P*. *vulgaris*, *S*. *aureus*, *Pseudomonas aeruginosa*, *E*. *coli*, *Bacillus megaterium*, *Sarcina lutea*, *K*. *pneumonia*, *A*. *niger*, and *C*.*albicans*. The findings of the study revealed that ZnONPs broke down cell walls, disrupted cell membranes, and ultimately resulted in cells dying. Not only did ZnONPs have antibacterial action, but they also showed biological effects against inflammation, antioxidants, diabetes, cancer, and aging [28].

A member of the Caesalpinioideae subfamily of the Fabaceae family is *Cassia javanica L*. There are seven subspecies of the polymorphic species *Cassia javanica L*. [29]. The young leaves have demonstrated their hypoglycemia and antioxidant properties earlier [30, 31]. According to Cheng et al.’s research, *C*. *javanica* exhibits HSV type-2 antiviral capabilities and is effective against influenza, constipation, stomach discomfort, and chickenpox. In Thailand, pyretic diseases are commonly treated at home using the bark along with seeds [32]. The existence of *C*. *javanica’s* bioactive phytoconstituents was confirmed by phytochemical analysis of ethyl acetate and methanol extracts of several portions of the plant, and sensitivity evaluations indicated that it is a strong antimicrobial agent [33]. *C*. *javanica* possesses a number of pharmacological characteristics including antibacterial, anticancer, antidiabetic, as well as antioxidant activity [34].

Current pharmacological research supports cassava plants. Notably, several actions have been discovered, including antioxidant, hepatoprotective, antimicrobial, anti-inflammatory, antidiabetic, hypolipidemic, and anti-atherosclerotic qualities. Numerous compounds, including anthraquinones, alkaloids, flavonoids, and other significant elements, are present in cassia species. Anthraquinones and anthracenes are the ones that show the maximum activity among them. To fill up the current information gaps about the phytochemistry and medicinal features of these important plants, more research is necessary [35].

The objective of this research was to generate ZnONPs in a green manner utilizing an aqueous extract of *Cassia javanica L* and assess the antibacterial, antibiofilm, antioxidant, antiviral, and anti-cancer properties. This is one of the few research that forms ZnONPs from *C*. *javanica* extract, investigates the characteristics of the ZnONPs, and looks into a range of medical uses.

## Materials and methods

### Materials

The study’s supply of Zn (CH3COO)2·2H2O zinc acetate dihydrate, was supplied by the company Sigma-A in St. Louis, USA. The additional chemical compounds, culture medium, as well as reagents utilized in this investigation had been classified as analytical, meaning no purification was necessary, they were acquired from Modern Lab Co., India.

### Preparation of *Cassia javanica L* extract for zinc oxide NPs synthesis

*Cassia javanica* tree cultivated in the garden of the Faculty of Science, Al-Azhar University. The healthy and disease free medicinal plant *Cassia javanica* leaves were collected during the month of November 2023, from the garden region of Faculty of Science, Al-Azhar University, Egypt following established protocol and permission was obtained. Further, the plant material was identified at the Department of Botany and Microbiology, Al-Azhar University, Egypt. *Cassia javanica* fresh leaves were collected and cleaned with Milli-Q sterile water. Following their cutting, these were then dried for approximately 24 hours at 60°C. With 200 milliliters of D. H2O, 10 grams of dried *Cassia javanica* plant were warmed. After that, it was, filtered through Whatman No. 1, and stored in the refrigerator until additional usage.

### Biosynthesis of zinc oxide NPs

The development of ZnONPs involved the utilization of the refined *Cassia javanica L* extract. The proper reaction mixture was prepared by mixing 50 mL of 0.1 M Zn(CH_3_COO)_2_·2H_2_O solution with 20 mL of the *Cassia javanica L* extract inside a clean flask. However, a control group of people employed the same experimental established substituting 50 mL of distilled water for 20 mL of *Cassia javanica L* extract. To generate a uniform mixture, the two flasks had been placed within a rotor shaker for 2 hours. Centrifugal force along with dist. water were used to remove impurities from the resulting ZnONPs [36]. To facilitate additional investigation and biological activity assessment, the dehydrated ZnONPs have been preserved at ambient temperature.

### Characterization of zinc oxide NPs

The duration of the incubation allowed for the visual observation of ZnONPs formation by alterations in mixture color. UV-Vis spectrum (JENWAY 6305 Spectrophotometer) had been used for investigating the generation of ZnO colloidal material by *Cassia javanica L* extract at wavelengths ranging from up to 800 nm. The bio produced zinc oxide NPs were examined using FTIR study in order to pinpoint functional groups crucial to the integrity and reduction of zinc oxide NPs, The FTIR spectrometer (Agilent Technology Carlyle 630 FT-IR version) as well as the potassium bromide (KBr) pellets method were used for the analysis. We acquired frequency measurements with a spatial accuracy of 4 cm^−1^ in an area of 4000–400 cm^−1^. The zinc oxide NPs were analyzed with an X-ray diffraction X’Pert Pro (Philips Electronics, the capital of Netherlands) that ran at 40 kV as well as 30 mA which had been fitted with a Ni-filter/Cu-K irradiation ratio detectors (=1.5405), zinc oxide NPs were examined for their crystalline nature at ranges between 10 and 80°C. The Joint Commission on Particle Diffraction Standards database was used in assigning and comparing the peak values (JCPDS). The chemical makeup of the generated ZnONPs was determined by EDX analysis. Utilizing TEM (JEOL 1010, Tokyo, Japan) to evaluate the size and shape of generated zinc oxide NPs. To generate the specimen, the zinc oxide NPs solution was poured upon a Cu-grid that had been covered with carbon and set on the specimen stage.

### Antibacterial activity

This investigation assessed the antibacterial efficacy of biosynthesised zinc oxide (NPs) against a set of five pathogenic microbial strains, including *Salmonella typhimurium* (ATCC 14028), *Escherichia coli* (ATCC 8739), *Clostridium sporogenes* (ATCC 1940), *Bacillus subtilis* (ATCC 6633) and *Bacillus pumilus* (ATCC 14884). Each strain of the microbes under investigation was uniformly distributed on sterile petri dishes after being cultured as a pure strain in MH broth. the agar diffusion well technique, which involves drilling a 7 mm circular well on plates using a clean cork borer. Each well received 100 μL of zinc oxide nanoparticles to assess the effectiveness against microorganisms. The plates were left to incubate at 37°C for the whole night, after which the inhibition areas were subsequently computed [37, 38]. A plant extract of *Cassia javanica L* had been carried out with beginning ingredient (zinc acetate) as a control. We incubated the plates at 37°C for twenty-four hours. Evaluating the circumference of a zone that grew clearly around each well as the incubation period came to an end was the next step.

The developed zinc oxide NPs antimicrobial efficacy towards the strains was evaluated using the broth-based microdilution method. Consequently, it made possible to calculate zinc oxide NPs minimal inhibitory concentration (MIC). Sequential dilution of zinc oxide NPs twice over was a part of this experiment. Ten microliters (μL) of pure microbial strains, or half of a McFarland standard, had been added to zinc oxide NPs. After that, the obtained specimens had been incubated around 37°C until 24 hours [38]. The MIC could be determined via a microplate reader (STATFAX, USA) and the lowest dosage of the test samples that suppressed the test microorganisms equivalent to positive and negative controls, in accordance with the Clinical and Laboratory Standards Institute (CLSI) guidelines [39]

### Anti biofilm activity

The capacity of zinc oxide NPs to prevent or decrease the biofilm accumulation that results from clinical species MRSA (Methicillin Resistant *S*. *aureus*) was assessed using the MTP technique. (found to be a strong biofilm-forming strain) subsequent to minor adjustments [40]. In summary, zinc oxide nanoparticles (NPs) were dispersed at variable dosages onto a flat-bottomed microtitre plate (MTP) that contained a tryptic soy broth medium (TSB) including 1% glucose. The planktonic bacteria were taken away from every well of the MTP holes avoiding any disturbance within the biofilm that had currently developed. The pathogenic bacteria that were being assessed was adjusted to obtain the size of the inoculum 1.5 × 108 CFU/mL, which was next transferred onto MTP along with kept at 37°C throughout 48 hours. In addition, those holes were multiple times rinsed with phosphate-buffered saline (PBS) in order to eliminate any remaining floating boundless cells. Methanol 95% had been added to each well to make a proportionate amount of 250 μL in order to assist in the establishment of biofilm. Subsequently, add 250 μL of diluted (0.3%) Crystal violet into the identical wells, and incubate the MTP approximately fifteen minutes at 25°C. Furthermore, sterilized distilled water was used to carefully remove any extra CV staining. To determine biofilm development quantitatively, 250 μL of 30% glacial acetic acid was then added to each well. Finally, the color was measured around 540 nm employing a Tecan Elx800 microplate analyzer. We contrasted the outcomes of the medicated versus untreated holes.

### Antioxidant assay of ZnONPs

The 1,1-diphenyl2-picryl hydrazyl (DPPH) technique has been employed to quantify the ability to neutralize free radicals of various concentrations of zinc oxide NPs in order to assess their effectiveness as antioxidants. To put it briefly, a 0.1 mM DPPH suspension with ethanol had been prepared. One milliliter of the resulting solution (1 mL) was mixed with three milliliters of various ZnONPs quantities from (1000 to 7.8 μg/mL) in ethanol made using the dilution procedure. After giving the chemical reaction combination containing DPPH and zinc oxide NPs a good shaking and letting it stand at 20°C for thirty minutes. The absorption value at 517 nm was obtained utilizing a spectrophotometer (UV–Vis Milton Roy). This set of tests used the antioxidant ascorbic acid to be the primary standard chemical. The zinc oxide NP dosage needed for inhibiting 50% of the DPPH free radical is known as the value of the IC50, and it was determined employing a log dosage inhibitory curve.

To calculate antioxidant activity, employ the following formula.

$$Antioxidant\;activity\;(\%)=\frac{A1–A2}{A1}X\;100$$

where A1: control absorbance

A2 test sample absorbance

### Cytotoxicity and antitumor activity in vitro

The cytotoxicity of generated ZnO-NPs versus normal Vero cell (ATCC CCL-81) cell lines and anti-cancer toward HepG2 (ATCC HB-8065) was evaluated using the MTT assay [41].

To form a full monolayer sheet, 100 μL/well of 1 × 105 cells/mL were added to a tissue culture plate (96 wells) and incubated for 24 hours at 37° C. Once a confluent sheet of cells formed, the growth media were eliminated from 96-well microtiter plates and each cell monolayer subsequently rinsed twice with washing media. In a medium made from RPMI containing 2% serum, the analyzed sample has been diluted two times (maintenance medium). Every solution was then examined for 0.1 mL in a separate well, leaving three wells as controls that contained just maintenance medium. After that, the plate was incubated at 37°C and looked at. We evaluated for physical indicators off cytotoxicity within the cells, such as shrinkage, rounded off, granulation, or either a partial or whole destruction of the cell monolayer. After making a 5 mg/mL solution of MTT (Bio Basic Inc., Canada), 20 μL of the resulting mixture was transferred to every well. The specimens were subsequently agitated for 5 minutes at 150 rpm, following which they were incubated for 4 hours at 37°C using 5% CO_2_ to enable the MTT to begin being metabolic. It was determined what the optical density was at 560 nm. We computed the percentages of cell viability and cell inhibition using the following Equations.

Viability % = OD of test/ OD of control X 100

Inhibition % = 100 − Viability %.

### Antiviral assay

Plate 10,000 cells employing 200 ul of water per well of a 96-well plate. To accommodate controls, three of the wells should remain empty. Set aside for the full overnight at 37°C with 5% CO_2_ to allow the cells to adhere within the plate holes. After one hour, incubate the specimen under examination at a harmless dose of power source at the same amount (1:1 v/v) with the viral suspension. Add 100 μl and let the viral/sample solution sit for a while. Place it on top of an unsteady table and spin at a speed of 150 rpm for a total of five minutes. To allow the virus to start operating, leave it for a day around 37°C and 5% CO_2_. Make 2 ml or more of the mixture employing 5 mg/ml of MTT in PBS for every 96-well plate. Put 20 ul from the MTT solution into each well. To completely integrate the MTT within the medium, place on a shaky surface along with rotate at 150 rpm for a duration of five minutes. Incubate the MTT reagent at 37°C using 5% CO_2_ for one to five hours to allow metabolism to occur. If necessary, remove any residues by emptying the medium (dry plate) with tissue paper. You can dissolve formazan, an MTT metabolic product, again in 200 μL DMSO. Place the formazan as well as solvent onto a shaker, then shake at 150 rpm to 5 minutes to fully mix them. Evaluate optical density at approximately 560 nm as well as eradicate interfering at 620 nm. There should be a clear correlation among the quantity of cells as well as optical density [42, 43].

## Results and discussion

### Generating a *Cassia javanica L* leaf extract

As Owoeye et al., with minor modifications, the gathered *Cassia javanica L* leaves were using Milli-Q sterile water to clean [44]. These were subsequently dried at 60°C for around 24 hours after they were cut. Ten grams of dried *Cassia javanica* plant were warmed in 200 milliliters of D. H2O. The filter paper from Whatmann No. 1 was used to filter the mixture after three hours in order to generate the aqueous leaf extract. Prior to being utilized in the synthesis of ZnO nanoparticles, the aqueous extract was refrigerated to 4°C.

### Bio-synthesis of zinc oxide NPs via *Cassia javanica L*

Plants are thought of as nature’s production facilities for chemicals because they are low-maintenance and affordable. Considering they generate huge amounts of phytochemicals, a variety of plant components, particularly fruit, leaves, stems, and roots, have been employed extensively for the environmentally friendly creation of nanoparticles [45]. Extracts from plants contain phytocompounds as polyols, terpenoids, and polyphenols that produce metallic ions through bioreduction [46]. For fifteen minutes at room temperature, approximately 0.1 M zinc acetate dihydrate was added to with 50 mL of deionized water while being continuously stirred. Next, 20 milliliters of an aqueous leaf extract solution were mixed with the zinc acetate solution. The resulting mixture was aggressively stirred in a magnetic stirrer for a full two hours at 65 °C. The resulting precipitate was given time to settle when the reaction was finished, and it took place. Centrifuging about 6000 rpm for fifteen minutes allowed the precipitate to be separated from the reaction solution. Any remaining contaminants were washed away in a series of deionized water washes before being dried at 200°C in an air oven. Following drying, the ZnO NPs had been crushed and kept in a container that was airtight. Fouda et al. state that combining the zinc acetate solution together with the aquatic extract of Punica granatum produced white ZnONPs [47]. Zinc oxide NPs and other NPs were produced in a green biosynthesis process employing Ziziphus spina-christi by Lashin et al [48].

### Characterization of ZnONPs

**Fig 1:**
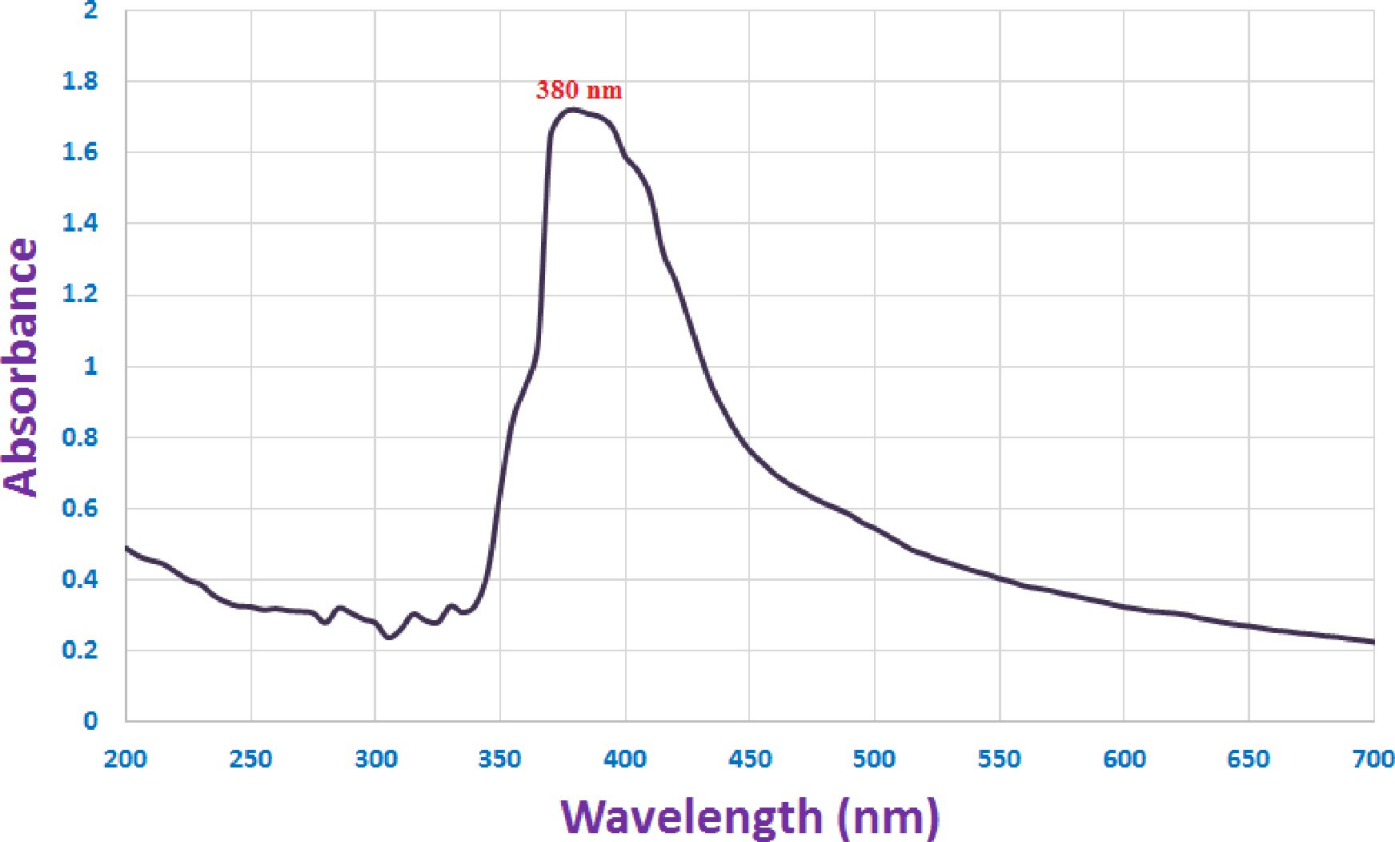
Uv spectrum of ZnONPs produced by *Cassia javanica*.

Zinc oxide nanoparticles are present when the color of the precipitate turns white. The absorbing capacity of the resultant color has been determined in the 200–700 nm region with the objective to determine the maximum surface plasmon resonance. The highest SPR for synthesized ZnO nanoparticles was observed at 380 nm over a 24-hour period **(Fig 1)**. After being biosynthesized from Vernonia cinerea leaf extract, the Zn- ONPs had an absorbance spectrum of 360 nm when exposed to UV [49]. ZnONP presence in the water-based solution was verified by the absorbance peak near the resonance wavelengths of 270 nm [50]. Ae-ZnONPs’ UV spectra show two distinct peaks at 275 as well as 380 nm, correspondingly [51]. Furthermore, the greatest SPR obtained at 380 nm indicates that the zinc acetate was effectively converted into its final form (ZnONPs) [52].

**Fig 2:**
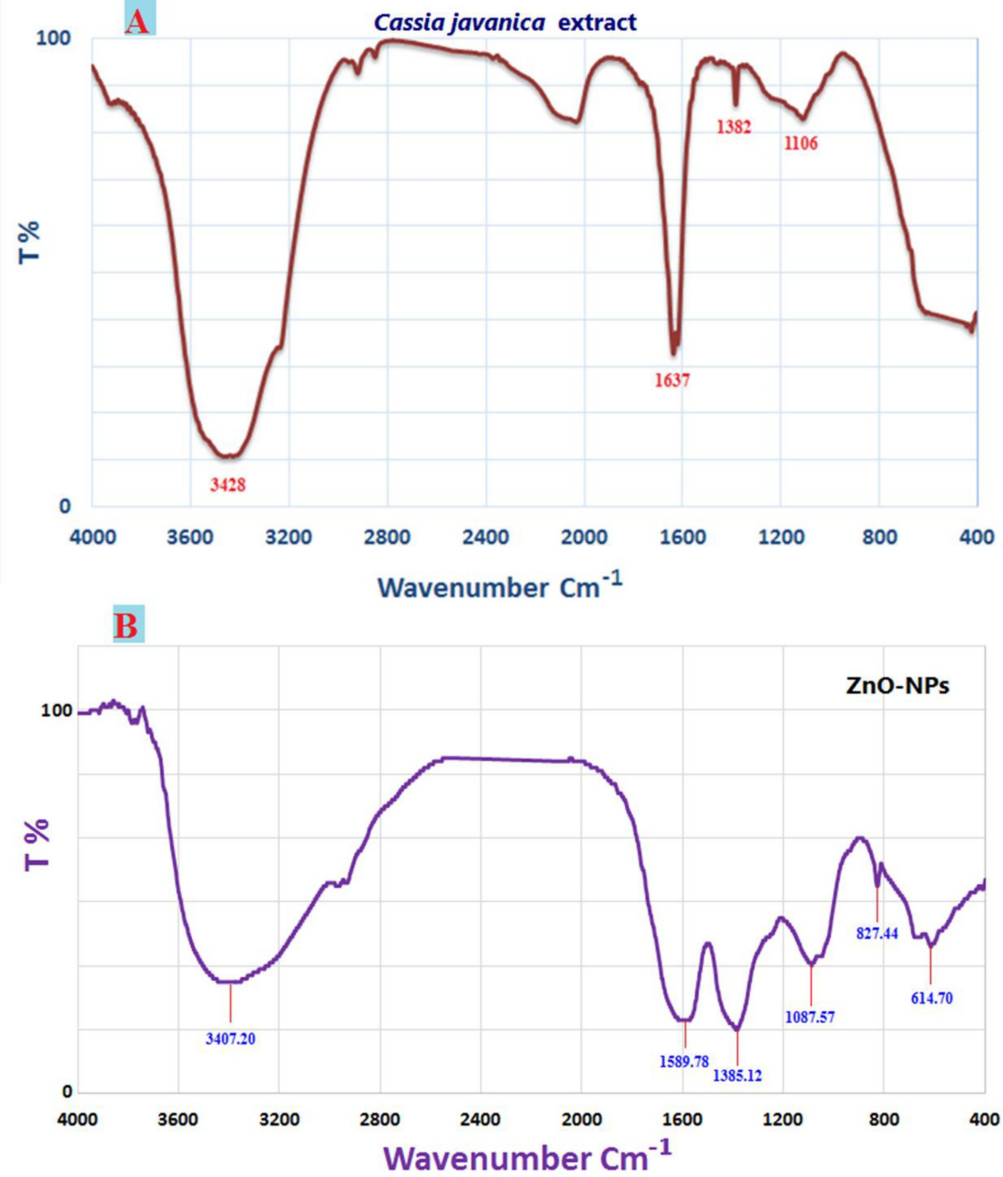
FTIR spectrum of *C*. *javanica* extract (A) and ZnONPs (B) produced by *Cassia javanica*.

The various functional groups found within the cell-free filtrate along with their roles in the synthesis and stabilization of ZnO NPs were identified using FT-IR analysis. As seen in **Fig 2A**, the plant extract FT-IR chart shows peaks at certain wave numbers, 3428, 1637, 1382, and 1106 cm−1. The ZnO NPs FT-IR chart displays peaks at certain wave numbers, 3407.20, 1589.78, 1385.12, 1087.57, 827.44, and 614.70 cm−1, as shown in **Fig 2B**. In contrast, C=O, C-N bonds with aromatic expanding, and C-O are linked to the maximum bands at 1589.78, 1385.12, 1087.57, and 827.44 cm−1 [53, 54]. Through biosynthesis, there were variations in the NPs’ peaks. At 400–700 cm−1 peaks. It was confirmed that ZnO NPs were successfully synthesized. The band at 614.70 cm−1 provides the final evidence of the zinc oxide bond. The data collected verified the presence of many functional groups, including aromatic and aliphatic amines, and alkyls, in *Cassia javanica L* leaf extract. These groups are essential for the reduction, capping, and stability of ZnONPs [55, 56]. Dias et al. reported findings, stating that FT-IR analysis revealed a prominent peak at 432 cm−1, indicating the distinctive Zn-O bond. Regarding the ZnO NP synthesized in an environmentally friendly manner [57].

**Fig 3:**
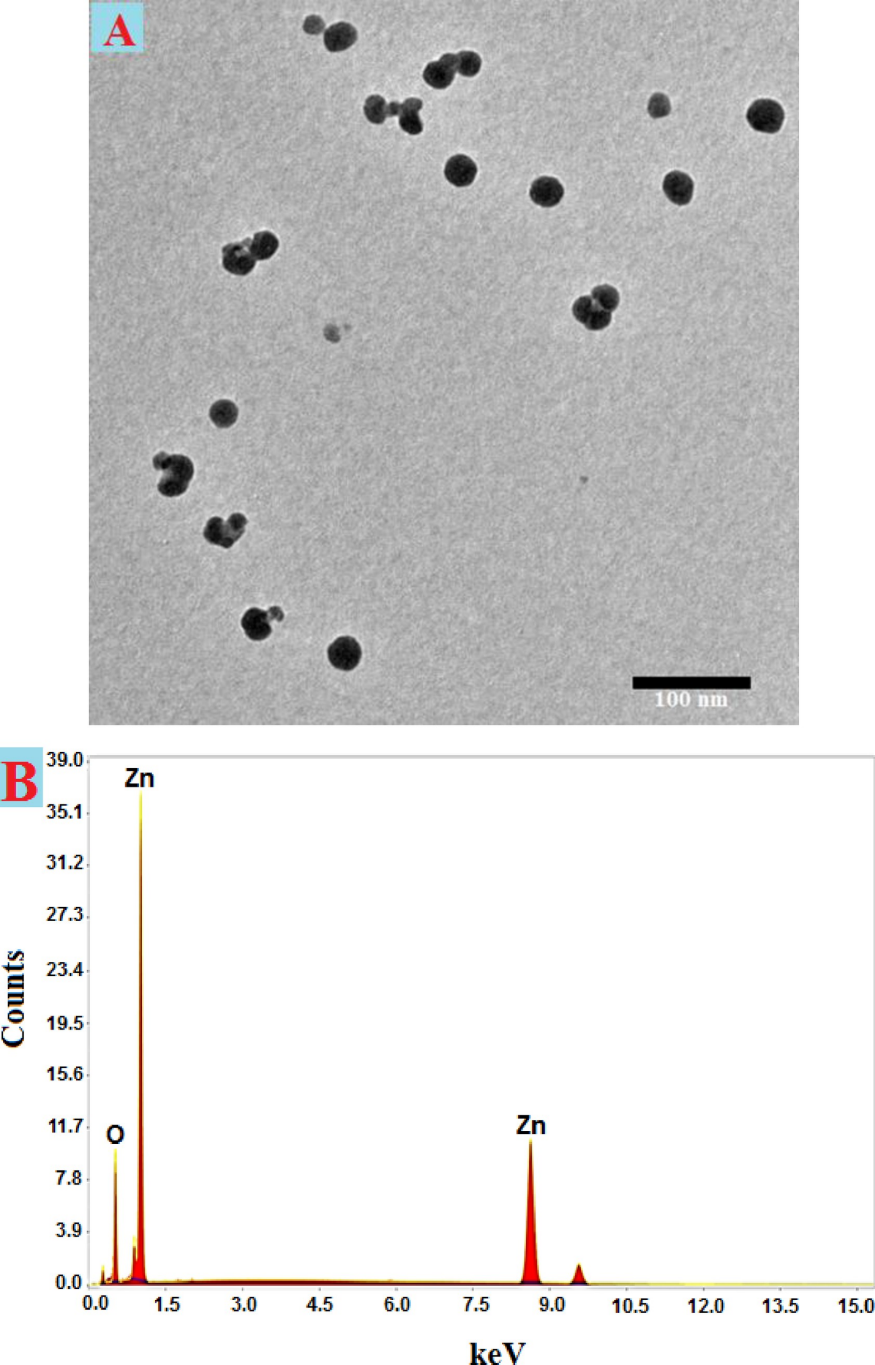
TEM image (A) and EDX analysis (B) of ZnONPs produced by *Cassia javanica*.

TEM is frequently exploited to examine the morphological characteristics of synthesized NPs, including their size, accumulation, and shape. As demonstrated, zinc acetate may be reduced or capped by phytochemicals generated by *Cassia javanica L*, resulting in spherical, widely dispersed ZnONPs (**Fig 3A**). The biosynthesized ZnONPs ranged in size from 15 to 40 nm. Utilizing the marine aquatic algae *Ulva fasciata Delile*, spherical ZnO nanoparticles having a mean size of 10.62 nm and a variety of sizes of 3–33 nm were effectively synthesized [58]. Furthermore, spherical ZnONPs featuring sizes that range from 6 nm to 21 nm were produced by Abdo et al. The electrostatic attraction of NPs can be influenced by a wide range of elements, including as surface characteristics, size, shape, coating or capping agent, responsiveness, and solubility [59]. It was reported that the spherical-shaped zinc oxide nanoparticles ranged in size from 28 to 42 nm was generated [60]. These results led us to believe that the low sizes of the produced ZnONPs employed in the current work will result in high reactivity. The chemical makeup of the generated ZnONPs was determined by EDX analysis, and the outcome is shown in **Fig 3B**. In our investigation, the EDX spectrum was used to detect the components of zinc and oxygen. According to the ZnONPs ingredient description, there is 22.55% oxygen and 77.35% zinc.

**Fig 4:**
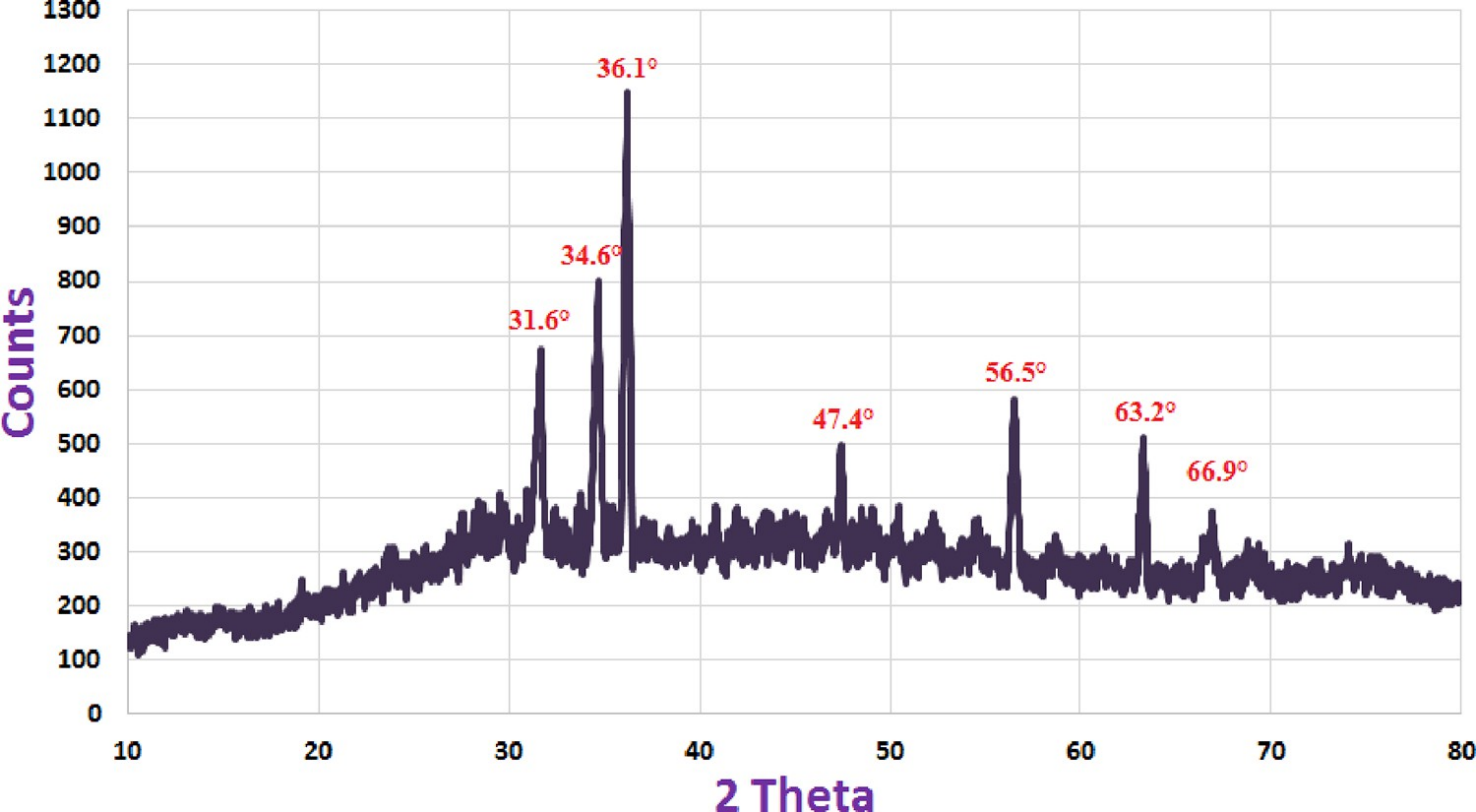
XRD pattern of ZnONPs produced by *Cassia javanica*.

ZnONPs that were biosynthesized and whose crystallinity fell between two theta values were investigated using XRD (**Fig 4**). At (100), (002), (101), (102), (110), (103), and (112), seven prominent peaks for Bragg reflection are visible in the spectra, with two theta values of 31.6°, 34.6°, 36.1°, 47.4°, 56.5°, 63.2°, and 66.9°, in that order. The results obtained demonstrated ZnONP’s crystallinity in agreement with the polycrystalline wurtzite form Diffraction Standards JCPDS data (JCPDS 5-0664) [61]. The results we found were supported by the XRD pattern, which indicated that the biologically generated ZnONPs were crystalline [52]. Lately, the various peaks roughly corresponded to the diffraction planes (101), (102), (101), (260), and (101). The results confirm the wurtzite composition of the biogenerated ZnONPs [49].

### Antimicrobial activity

**Fig 5:**
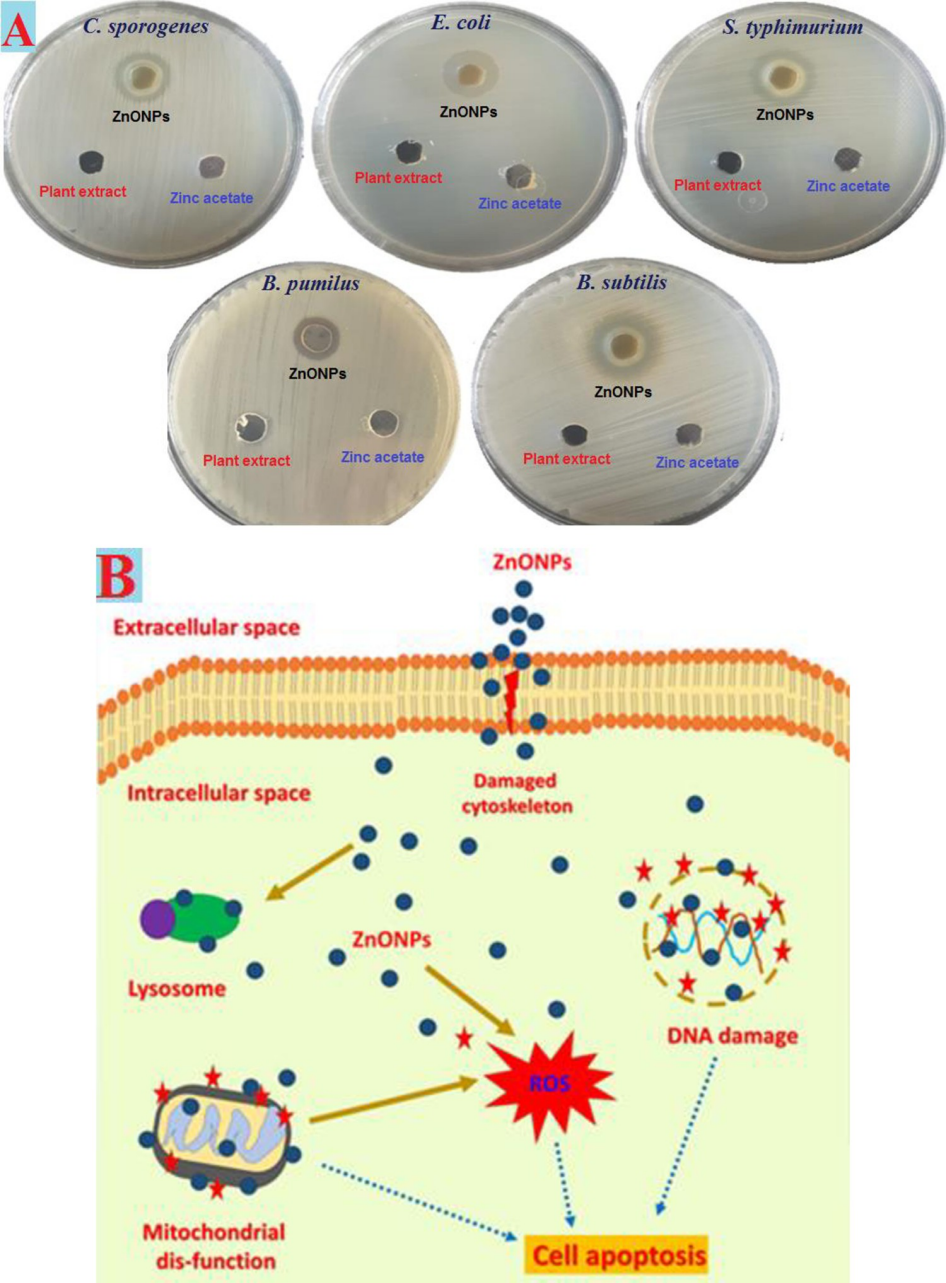
Antimicrobial activity (A) and mechanism of action (B) for ZnONPs.

Five pathogenic bacterial starins have been used to evaluate the antibacterial qualities of ZnONPs made from *Cassia javanica L* extracts. Zone of inhibition against microbiological infections was computed with 1000 μg/ml of produced ZnONPs and zinc acetate. On the other hand, biosynthesized ZnONPs demonstrated potent antibacterial activity comparable to that of the plant leaves extract. Zones of inhibition for ZnONPs’ antibacterial activity ranged from 15 to 19 mm **(Fig 5A)**. In the current study, ZnONPs produced using *Cassia javanica L* show substantial antibacterial effectiveness against bacterial strains (S1 Table). Compared to Gram-positive bacteria, which have thick peptidoglycan containing an inner phospholipid covering, gram-negative bacteria have cell walls which are rich in lipopolysaccharides and have a thin coating of peptidoglycan. ZnONPs are especially effective against Gram-negative bacteria, therefore this enhances their ability to destroy infections [58, 62]. An electrostatic interaction that occurs between the negatively charged lipopolysaccharide and the highly charged ZnONPs may be the cause of the greater suppressive effect seen against Gram-negative bacteria [62]. Some of the mechanisms behind the antibacterial action of zinc nanoparticles are explained **(Fig 5B)**, including how they impact the cell wall before penetrating the cell and damaging it. It stops energy generation and prevents the creation of DNA by destroying mitochondria and protein synthesis through its action on free radicals. Ultimately, the cell perishes and is destroyed [63, 64].

**Fig 6:**
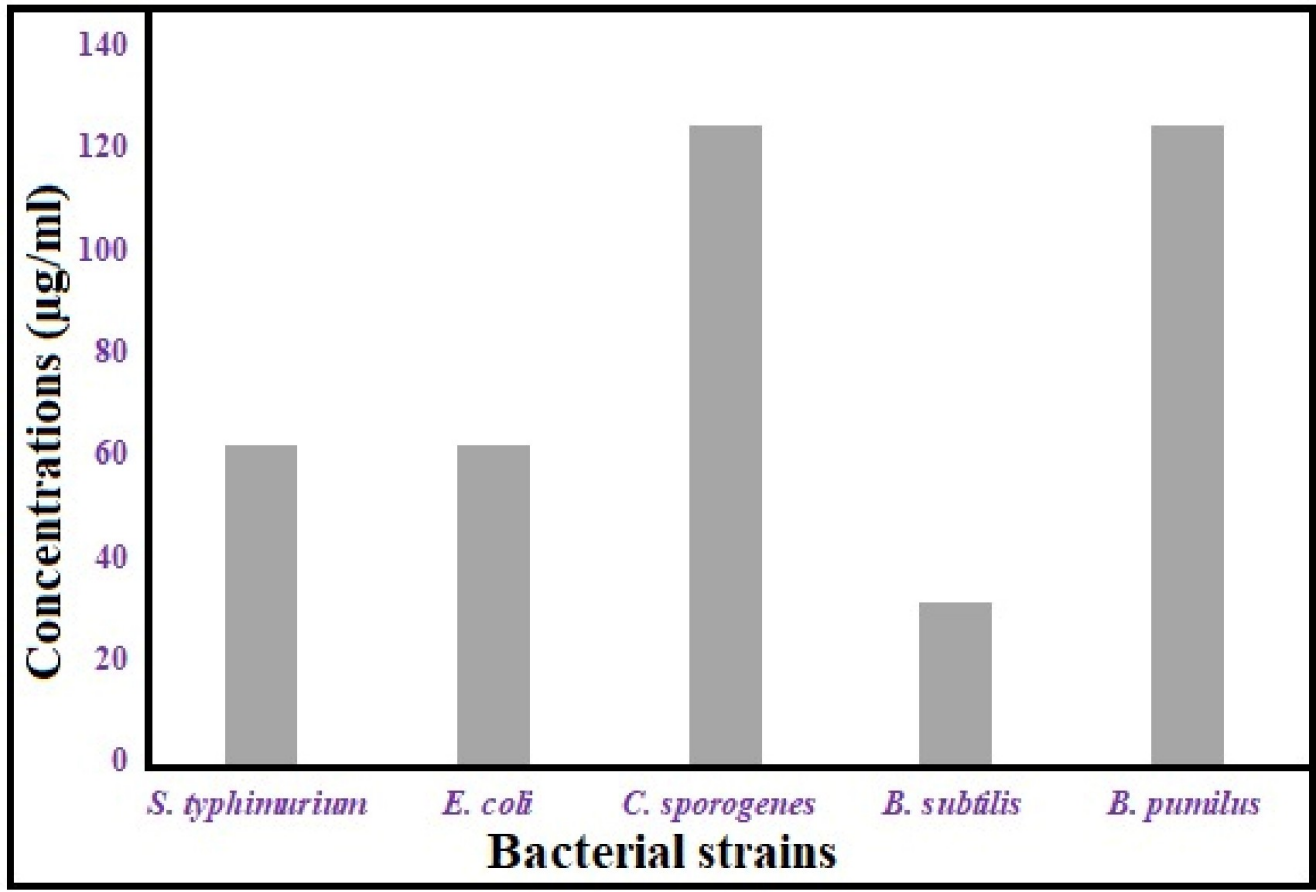
MIC for bacterial strains treated with ZnONPs.

ZnONP MICs were determined in relation to ATCC 14028 *Salmonella typhimurium*, ATCC 8739 *Escherichia coli*, ATCC 1940 *Clostridium sporogenes*, ATCC 6633 *Bacillus subtilis*, and ATCC 14884 *Bacillus pumilus* (S2 Table). The study examined the inhibitory impact of varying ZnONP concentrations (16.62-1000 μg/mL). The lowest MIC was 31.7 μg/mL for *B*. *subtilis*, while the MIC for *S*. *typhimurium* and *E*. *coli* was 62.5 μg/mL. The results indicated that the MIC for ZnONPs was 125 μg/mL against *B*. *pumilus* and *C*. *sporogenes* **(Fig 6)**.

Spherical-shaped ZnONPs (33 nm) showed significant antibacterial efficacy against both *E*. *coli* and *E*. *faecalis*. The antibacterial activity of naturally occurring ZnONPs made with different biological ingredients varied [65]. ZnONPs produced by cyanobacteria Oscillatoria sp. extraction shown dose-dependent inhibition versus MDR bacterial strains (*K*. *pneumoniae*, *E*. *coli*, *S*. *aureus* and *B*. *cereus*), with MIC values ranging from 62.5 to 125 μg mL^−1^. This was in contrast to ZnONPs produced traditionally [66]. ZnONPs (44.5 nm) with a spherical form were produced with *Pseudomonas putida*; *Bacillus cereus*, *Enterococcus faecalis*, *Acinetobacter baumannii*, *Pseudomonas otitidis*, and *Enterococcus faecalis* are among the bacteria that they exhibit antibacterial action against [67]. **Table 1** presents a comparison of our work with other studies with respect to several biological applications.

**Table 1:** The biological activity of ZnO nanoparticles.

Biological-mediated synthesize	Biological activity of ZnONPs	Ref.
*Bacillus megaterium*	Antibacterial activity (*H*. *pylori*)	[68]
*Bacillus licheniformis*	Antibacterial activity *(P*. *aeruginosa*, *B*. *subtilis B*. *pumilus*, and *Proteus vulgaris*)	[69]
*Cassia fistula*	Antibacterial activity (*K*. *aerogenes*, *E*. *coli*, and	[70]
	*Plasmodium desmolyticum)*	
*Boerhavia diffusa*	Antibacterial activity (MRSA)	[71]
*Sechium edule*	Antibacterial activity (*B*. *subtilis and K*. *pneumonia*)	[72]
*Azadirachta indica*	Antibacterial activity (*K*. *aerogenes* and *S*. *aureus)*	[73]
*Bauhinia tomentosa*	Antibacterial activity:	[74]
	• *E*. *coli • P*. *aeruginosa*	
*Pichia kudriavzevii yeast*	Anticancer activity: MCF-7, breast	[75]
*Aspergillus niger*	Anticancer activity: HepG2	[76]
*Aspergillus terreus*	Anticancer activity: MCF-7	[77]
*Sargassum muticum*	Anticancer activity: WEHI-3, murine leukemia	[78]
*Costus pictus*	Anticancer activity: DLA, Daltons lymphoma ascites	[79]
*Manginefa*. *indica* [Mango]	Antioxidant activity	[80]
*Suaeda aegyptiaca* [Sea blit]	Antioxidant activity	[81]
*Costus igneus* [Spiral flag]	Antibiofilm, antidiabetic, and antioxidant	[82]

### Anti-biofilm assay of ZnONPs

**Fig 7:**
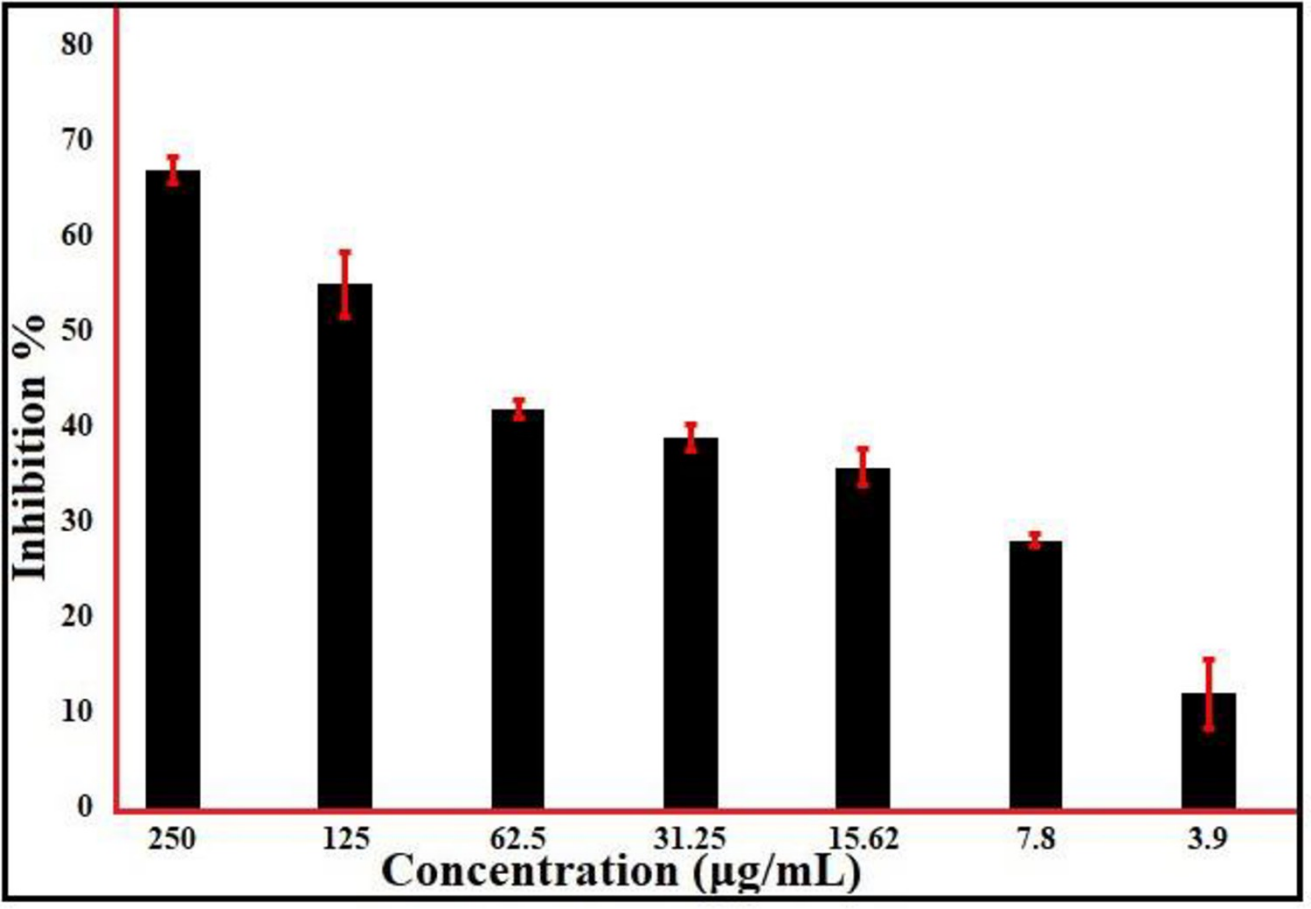
Antibiofilm assay of phyto synthesized ZnO-NPs.

In the current study, ZnONPs’ antibiofilm activity against MRSA, a biofilm producer bacterium, exhibited a variety of results (S3 Table). Consequently, ZnONPs showed the maximum suppression of the formation of S. aureus biofilms without sacrificing bacterial viability, with inhibition reaching 67.48% at 250 μg/mL and 12.48% at 3.9 μg/mL when used at concentrations below the MIC value **(Fig 7)**. Assessments, both qualitative and quantitative, demonstrate that ZnONPs prevented S. aureus biofilm formation during the first stage. In a separate investigation, biofilm development was inhibited during the irreversible adhesion stage by naturally occurring metal oxides. It’s important to remember because the initial biofilm development was suppressed at the MIC values [83]. Another research examined how metal oxide nanoparticles (NPs) might induce oxidative stress and communicate with microbial cell membranes to decrease biofilms [84]. Previous research elucidated the anti-biofilm action using SEM images, which demonstrated the existence of bacterial cell breaking, outer cell the degree of hardness as well as inner wall shrinkage. Less biofilm formation and survival of cells were also seen [85].

Lower minimal biofilm inhibitory concentrations (MBIC) of 46.8 μg /ml and 93.7 μg/ mL were observed in ZnONPs generated utilizing cyanobacteria cell extract, which demonstrated increased antibiofilm action versus MDR bacteria (S. *aureus*, *K*. *pneumoniae*, *B*. *cereus* and *E*. *coli*) [86]. ZnONPs (19.8 nm) which were produced via the unripe fruit extract of *A*. *marmellos* had improved antioxidant, antibacterial, and antibiofilm properties. Moreover, KC-ZNONPs, which are ZnONPs wrapped in cellulose, had improved anti-inflammatory and antibacterial efficacy versus MRSA strains and were biocompatible with human red blood cells [87].

### Antioxidant activity

**Fig 8:**
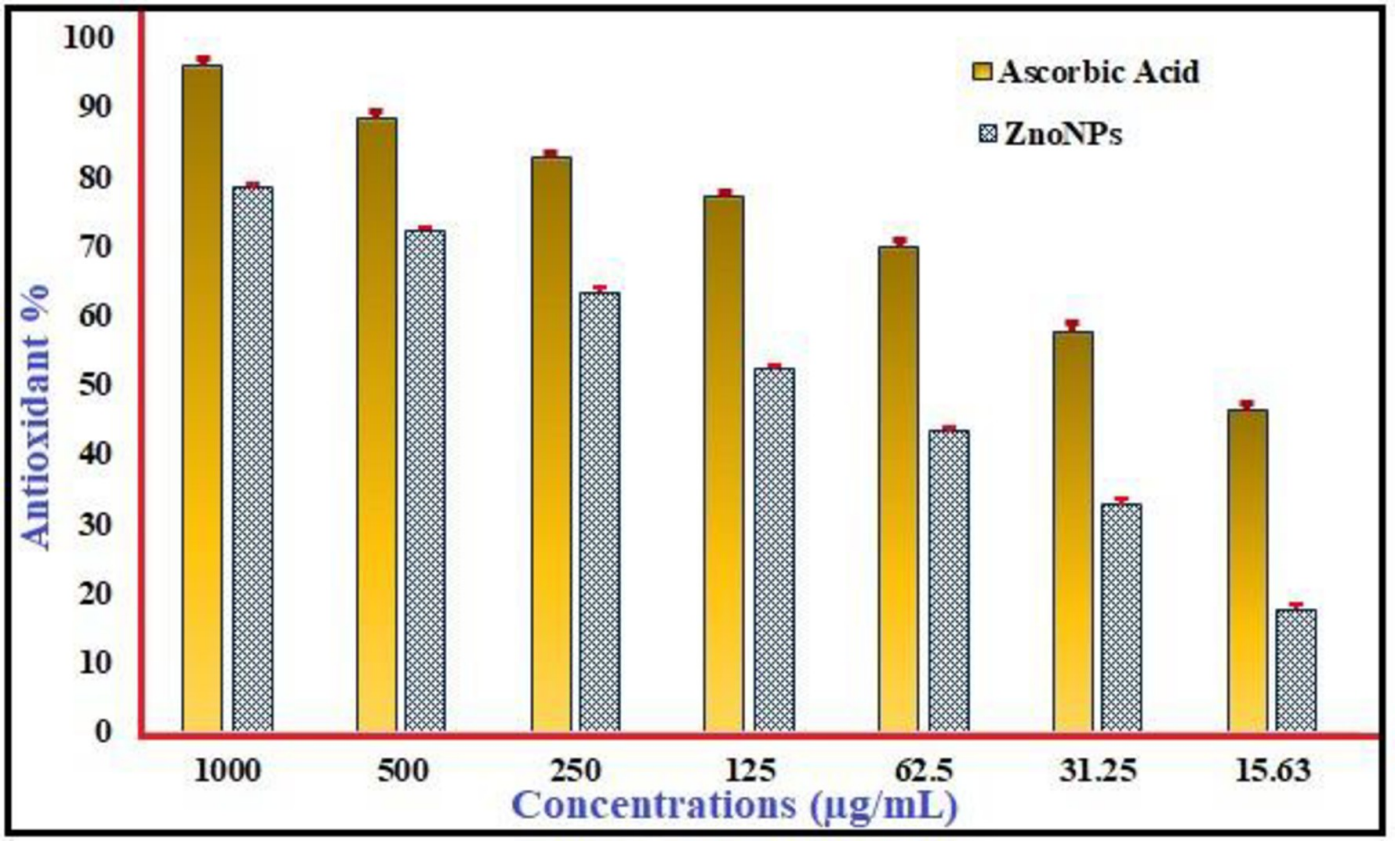
Antioxidant assay of phyto fabricated ZnONPs.

The DPPH method was used to evaluate the antioxidant capabilities of ZnONPs. ZnONPs showed a moderate level of DPPH radical-scavenging activity, as seen in **Fig 8**, as shown was measured at absorbency at 517 nm, which was utilized to determine the color shift from purple to yellow. ZnONPs showed 78.446% inhibition at 1000 μg/mL and 17.64% at 15.62 μg/mL. Compared to the standard, ascorbic acid was determined to be 96.26% at 1000 μg/ml, but only 46.49% at 15.62 μg/mL (S4 Table). ZnONPs’ radical-scavenging capacity was frequently less than comparable to ascorbic acid; the above findings imply that ZnONPs’ radical scavenging activity is dose-dependent [88]. Naturally occurring ZnONPs were shown to have a great capacity to scavenge radicals like DPPH; an IC50 value of 109.3 μg/mL was achieved with an effective antioxidant dose. The antioxidant activity of ZnONPs could possibly be attributed to the polyphenolic compounds that remained within the outer layer; these compounds that are bioactive aid in the donation of hydrogen atoms, allowing DPPH to transform to its less concentrated state [89]. Another research (DPPH) validated ZnONPs’ capacity to scavenge superoxide and hydrogen peroxide [90]. Oscillatoria-derived ZnONPs showed much higher antioxidant abilities than ZnONPs produced cheaply; they exhibited an inhibitory concentration of 50 for 54.2 μg mL^−1^ with an ABTS scavenging capacity ranging from 0 to 90%. Ascorbic acid induced restriction at 54.2 μg/mL [66]. ZnONPs made from Camellia sinensis showed their antioxidant potential in a particular kind of cell by functioning as cytoprotectants and successfully preventing H_2_O_2_-induced oxidative damage (3T3L1) [91].

### Cytotoxic and anticancer activity

**Fig 9:**
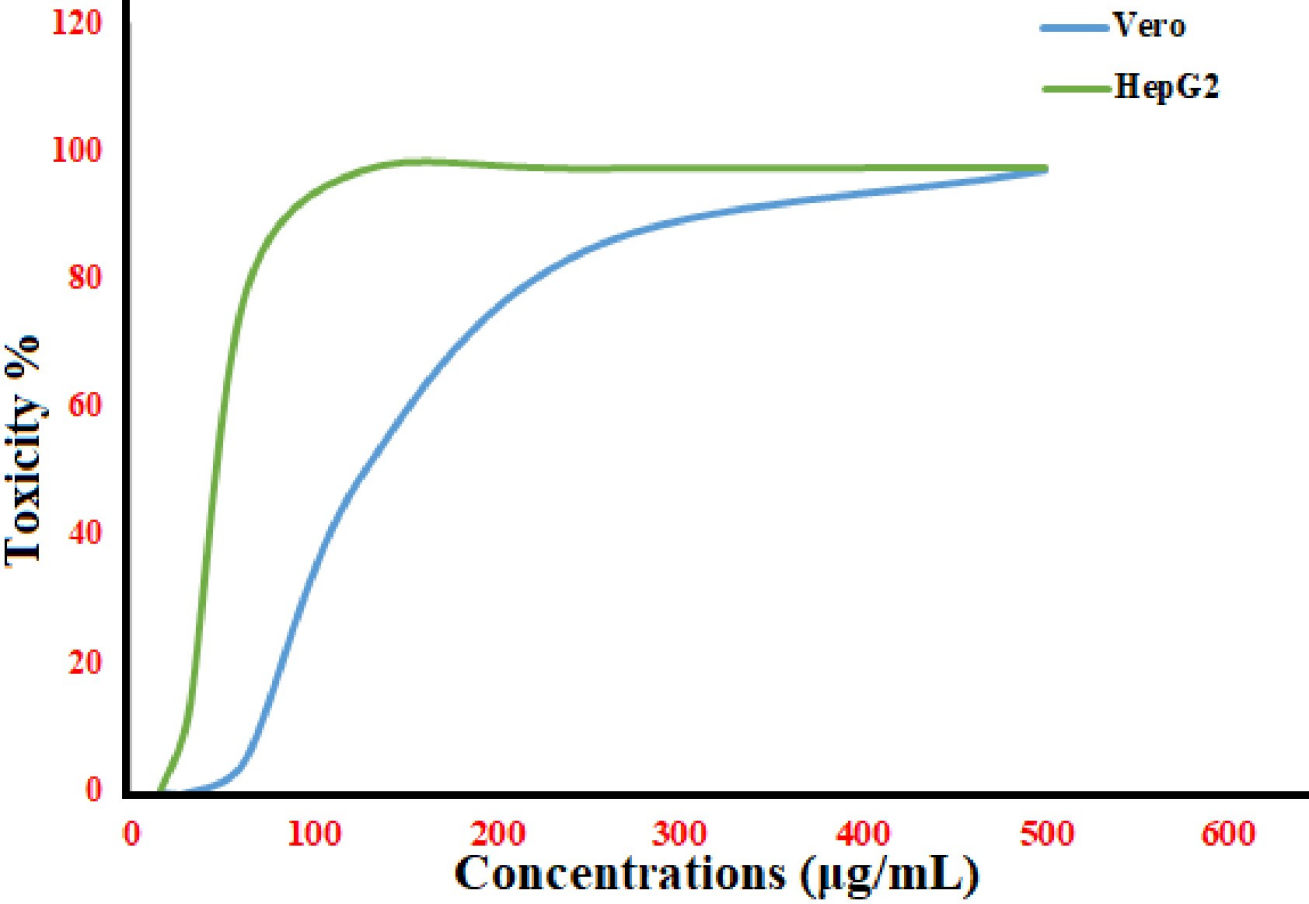
Cytotoxicity and anti cancer effect of ZnONPs.

The first step in evaluating the safety of biological materials is to assess their experimental cytotoxicity against kinds of normal cells [92]. Zn ionization to Zn ^2+^ as well as the generation of free radicals produced by the NPs face are known to be linked to ZnO-NPs toxicity, which causes a metabolic or ionic imbalance within the cell [93, 94]. The green synthetic zinc oxide nanoparticles (NPs) applied to the Vero cell line are shown in **Fig 9**. The median value (IC50) for nano-sized ZnONPs was determined to be between 500 and 15.62 μg/mL. The IC50 of the phytosynthesised ZnONPs was 154.01 μg/mL, based on the results (S5 Table). If the IC50 value of an ingredient is greater than 90 μg/mL, it is generally regarded as non-cytotoxic [95]. The cytotoxic impact of green synthetic ZnO was investigated using the MTT assay on the Vero, PK15, and MDBK cell lines at varied time intervals and concentrations. All types of cells showed improved cell viability with ZnO NPs at lower concentrations (10 μg/100 μL) [96]. ZnO NPs (10–50 nm) were demonstrated to have cytotoxic and genotoxic impacts on rat kidney epithelial cells during an additional investigation. The cytotoxic impact on NRK-52E kidney cell of rat was assessed using a range of assays, including Trypan Blue dye, MTT, and NRU at exposures concentrations of 25–100 μg/ml. The average of the IC50 levels was determined [97].

**Fig 10:**
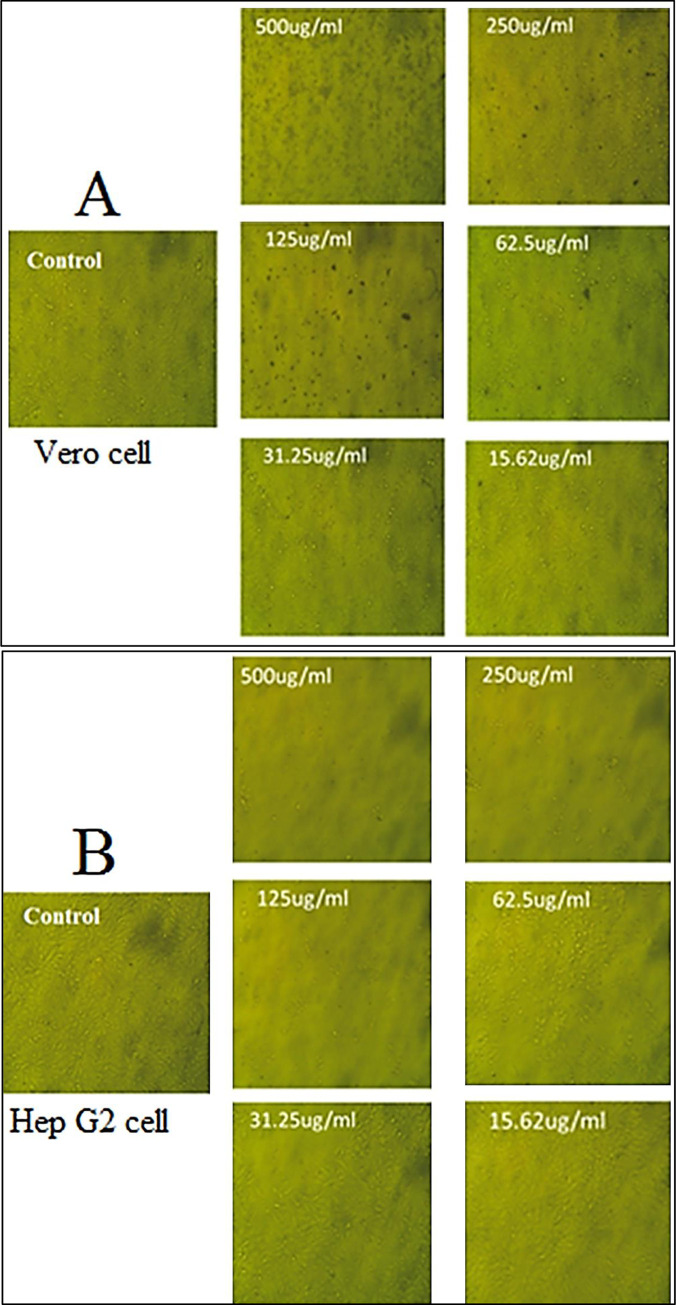
Vero cell (A) and Hep G2 cell (B) after exposure to ZnONPs by inverted microscope.

Various human cell lines, such as hepatocytes, kidney, and alveolar adenocarcinomas, have demonstrated the cytotoxic effects of ZnO NPs, dependent on the particle size and dose of the NPs employed [98, 99].We tested the anticancer properties of green fabricated ZnONPs towards cancer cells (HepG2) at doses ranging from 500 to 15.62 μg/mL (S6 Table). (**Fig 10**) displays the human hepatocellular carcinoma (HepG2. The findings showed that ZnONPs, with an IC50 of 47.48 μg/mL. Studies have also shown that *C*. *argentea* extract may be utilized as a reducing agent for ZnO NPs production with excellent selectivity for cancer cells with enhanced activity against cancer, supporting earlier research on the anticancer potential of biogenic ZnO NPs [100, 101]. In comparison with the alternative chemical ZnO NPs, it has been demonstrated revealed the green ZnO NPs had a greater percentage of cell viability in HepG-2 cells after a 24-hour exposure period. Furthermore, the highest percentage of cell viability loss was seen when green ZnO NPs were present at an amount of 120 μg/mL [102]. Furthermore, it is noticeable that healthy cells behave persistently in the green ZnO NPs, retaining at least 80% of their viability even in the face of increasing green ZnO NP formation [103].

### Antiviral activity

**Fig 11:**
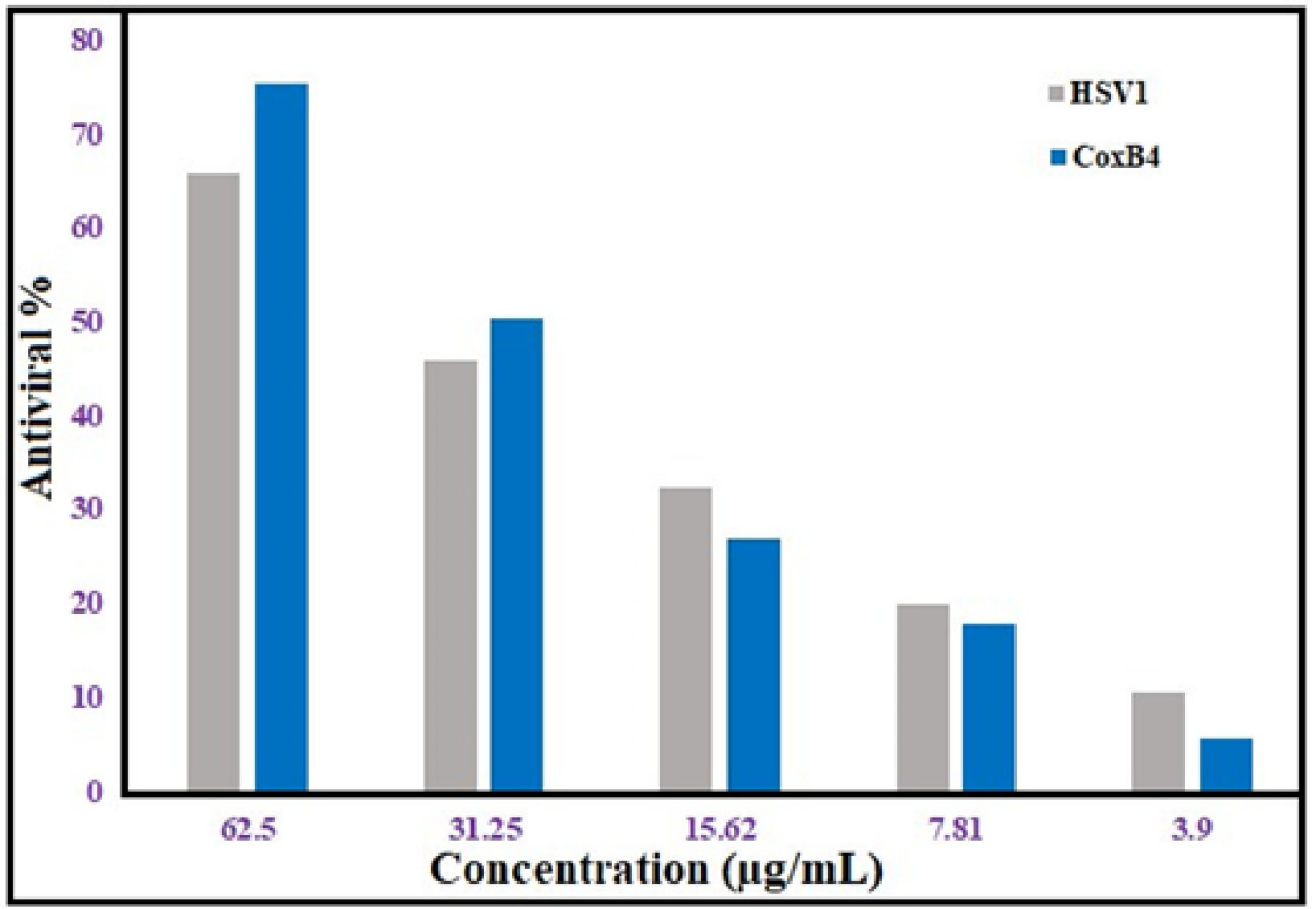
Antiviral activity of ZnONPs versus HSV1 and COX B4 viruses.

The effectiveness of ZnONPs in suppressing the development of the HSV1 and CoxB4 viruses was assessed in this study. Initially, the cytotoxic ability of ZnONPs against normal cell lines, was examined in order to identify the MNTC, which is 31.25 μg/mL. ZnONPs were discovered to have an attractive antiviral effectiveness against both HSV1 and COX B4. At the same dosage, COX B4 exhibited more activity than HSV1. Moreover, ZnONPs demonstrated 75.4% antiviral activity against COXB4, but 65.8% efficacy against HSV1 at 62.5 μg/mL **(Fig 11)**. Here, ZnONPs exhibit promising antiviral activity against both HSV1 and COX B4, indicating their suitability for use in biological fields. HSV1 is an example of a persistent infection (S7 Table). The primary therapy is acyclovir, although novel approaches to therapy are increasingly required due to the emergence of resistant strains. ZnO NP-based therapeutics are gaining a lot of attention. ZnO-NPs protect cultured corneas from HSV1 [104]. ZnONPs, which were made with an alcoholic extract of Plumbago indica, have been tested in vitro for their ability to damage HSV-1 grown on Vero cells. With an IC50 of around 23.16 μg/mL, HSV-1 proliferation is modestly suppressed [105]. Our findings corresponded with previous research that shown ZnO NPs exhibited strong antiviral activity starting at low concentrations (IC50 = 526 μg /mL), however they also had strong cytotoxic effects on the host-cell (IC50 = 292.2 μg /ml) [106]. NPs’ antiviral effectiveness has been explained by a variety of mechanisms, including interactions among viruses and cells that hinder infection, interactions between NPs and particular cell surfaces or sensors that prevent virus entry, inhibition of virus replication, prevention of viral spread, enhancement of oxidative stress via ROS production, cell apoptosis, and enhancement of the host cell’s immune response [107]. ZnONPs’ antiviral effect against HSV-1 may result from direct interactions between the virus parts, which trap the viruses and obstruct the virus’s ability to enter target cells. Another potential mechanism could include the selectively inhibitory impact of zinc ions on viral DNA polymerase, which inhibits HSV replication [108].

## Conclusion

The current study investigated the significant attributes of ZnO NPs and also explored their competence in various applications. This study examined environmental friendly biosynthesis of ZnO NPs via *Cassia javanica*. Different characterization techniques were employed to confirm the effective synthesis of ZnO NPs, which were further assessed for their diverse biological activities. The susceptibility of pathogens against these nanoparticles was also noticed with MICs of 31.7 μg/mL and 125μg/mL for *Bacillus subtilis* and *Bacillus pumilus*, respectively, while *Salmonella typhimurium*, *Escherichia coli*, and *Clostridium sporogenes* were 62.5 μg/mL along their biofilm inhibition potential. They also exhibit antioxidant properties and the ability to scavenge free radicals and IC_50_ was 109.3 μg/ml. However, their highest anticancer impact was seen against HepG2 cancer cell lines and IC_50_ was 47.48 μg/mL. Finally, ZnONPs exhibit promising antiviral activity against both HSV1 and COX B4, indicating their suitability for use in biological fields.

## Supporting information

S1 Table Antimicrobial activity of phyto-synthesized ZnO-NPs. (PDF)

S2 Table MIC for bacterial strains treated with ZnONPs. (PDF)

S3 Table Antibiofilm assay of phyto synthesized ZnO-NPs. (PDF)

S4 Table Antioxidant assay of phyto fabricated ZnONPs. (PDF)

S5 Table Viability and toxicity percent for Vero cells treated with different concentration of ZnO-NPs. (PDF)

S6 Table Viability and toxicity percent for HepG2 cells treated with different concentration of ZnO-NPs. (PDF)

S7 Table Antiviral activity of zinc oxide versus HSV1 and COX B4 viruses. (PDF)
