# Supplementary material for: Investigating the in vitro antibacterial, antibiofilm, antioxidant, anticancer and antiviral activities of zinc oxide nanoparticles biofabricated from Cassia javanica
Source: PLoS One. 2024 Oct 1;19(10):e0310927. doi: 10.1371/journal.pone.0310927 (PMC11444386; doi:10.1371/journal.pone.0310927)
Supplement: S1 Table — (PDF) [file pone.0310927.s001.pdf]

S1 Table: Antimicrobial activity of Phyto-synthesized ZnO-NPs.

Bacterial strains	R1	R2	R3	Mean	Std
S. typhimurium	18	17	17.5	17.5	0.5
E. coli	17.3	16.5	15.8	16.53	0.75
C. sporogenes	15.8	15	15.2	15.33	0.41
B. subtilis	18.5	18.9	19.3	18.9	0.4
B. pumilus	15	15.5	16.2	15.56	0.60
