# Supplementary material for: Investigating the in vitro antibacterial, antibiofilm, antioxidant, anticancer and antiviral activities of zinc oxide nanoparticles biofabricated from Cassia javanica
Source: PLoS One. 2024 Oct 1;19(10):e0310927. doi: 10.1371/journal.pone.0310927 (PMC11444386; doi:10.1371/journal.pone.0310927)
Supplement: S2 Table — (PDF) [file pone.0310927.s002.pdf]

S2 Table: MIC for bacterial strains treated with ZnONPs.

Bacterial strains	MIC
Salmonella typhimurium	62.5
Escherichia coli	62.5
Clostridium sporogenes	125
Bacillus subtilis	31.7
Bacillus pumilus	125
