# Supplementary material for: Investigating the in vitro antibacterial, antibiofilm, antioxidant, anticancer and antiviral activities of zinc oxide nanoparticles biofabricated from Cassia javanica
Source: PLoS One. 2024 Oct 1;19(10):e0310927. doi: 10.1371/journal.pone.0310927 (PMC11444386; doi:10.1371/journal.pone.0310927)
Supplement: S3 Table — (PDF) [file pone.0310927.s003.pdf]

S3 Table: Antibiofilm assay of Phyto synthesized ZnO-NPs.

Bacterial strains	R1	R2	R3	Mean	Std
ZnONPs			Inhibition %		
Conc (µg/mL)	R1	R2	R3	Mean	Std
250	67.27	66.18	69.01	67.4867	1.42739
125	52.72	54.54	59.27	55.51	3.38102
62.5	43.27	42.54	41.45	42.42	0.91591
31.25	38.9	38.54	41.09	39.51	1.38011
15.62	34.18	37.9	36.72	36.2667	1.90098
7.8	28.81	29.1	28	28.6367	0.57012
3.9	16.36	11.63	9.45	12.48	3.53255
