# Supplementary material for: Investigating the in vitro antibacterial, antibiofilm, antioxidant, anticancer and antiviral activities of zinc oxide nanoparticles biofabricated from Cassia javanica
Source: PLoS One. 2024 Oct 1;19(10):e0310927. doi: 10.1371/journal.pone.0310927 (PMC11444386; doi:10.1371/journal.pone.0310927)
Supplement: S4 Table — (PDF) [file pone.0310927.s004.pdf]

S4 Table: Antioxidant assay of Phyto fabricated ZnONPs.

	Antioxidant %				
**Ascorbic**	R1	R2	R3	Mean	Std
1000	95.8	95.68	97.32	96.26666667	0.914184518
500	88.63	87.5	89.5	88.54333333	1.002812711
250	83.65	82.75	82.65	83.01666667	0.550757055
125	77.96	77.21	76.32	77.16333333	0.820995331
62.5	69.14	69.86	70.9	69.96666667	0.884835201
31.25	57.9	56.2	58.9	57.66666667	1.365039682
15.63	45.76	46.12	47.6	46.49333333	0.975158107
**ZnO-NPs**					
1000	78.96	78.42	77.96	78.44666667	0.500533049
500	72.75	71.89	72.65	72.43	0.470319041
250	62.73	63.74	63.82	63.43	0.607536007
125	52.96	51.76	51.99	52.23666667	0.636893502
62.5	43.91	43.21	42.87	43.33	0.530282943
31.25	33.56	32.74	31.96	32.75333333	0.800083329
15.63	18.42	17.42	17.1	17.64666667	0.688573404
