# Supplementary material for: Investigating the in vitro antibacterial, antibiofilm, antioxidant, anticancer and antiviral activities of zinc oxide nanoparticles biofabricated from Cassia javanica
Source: PLoS One. 2024 Oct 1;19(10):e0310927. doi: 10.1371/journal.pone.0310927 (PMC11444386; doi:10.1371/journal.pone.0310927)
Supplement: S5 Table — (PDF) [file pone.0310927.s005.pdf]

S5 Table: Viability and toxicity percent for Vero cells treated with different concentration of ZnO-NPs.

ID	ug/ml	O.D			Mean O.D	±SE	Viability %	Toxicity %	IC50 ± SD
Vero	--------	0.713	0.72	0.718	0.717	0.002082	100	0	ug
ZnONPs	500	0.022	0.018	0.02	0.02	0.001155	2.789400279	97.21059972	154.01
	250	0.11	0.098	0.117	0.108333	0.005548	15.10925151	84.89074849	± 1.72
	125	0.352	0.369	0.371	0.364	0.006028	50.76708508	49.23291492	
	62.5	0.684	0.669	0.684	0.679	0.005	94.70013947	5.29986053	
	31.25	0.716	0.718	0.716	0.716667	0.000667	99.95351	0.046490005	
	15.62	0.714	0.718	0.719	0.717	0.001528	100	0	
