# Supplementary material for: Investigating the in vitro antibacterial, antibiofilm, antioxidant, anticancer and antiviral activities of zinc oxide nanoparticles biofabricated from Cassia javanica
Source: PLoS One. 2024 Oct 1;19(10):e0310927. doi: 10.1371/journal.pone.0310927 (PMC11444386; doi:10.1371/journal.pone.0310927)
Supplement: S6 Table — (PDF) [file pone.0310927.s006.pdf]

S6 Table: Viability and toxicity percent for HepG2 cells treated with different concentration of ZnO-NPs.

ID	ug/ml	O.D			Mean O.D	±SE	Viability %	Toxicity %	IC50 ± SD ug
HepG2	--------	0.75	0.745	0.74	0.745	0.002887	100	0	
ZnONPs	500	0.018	0.019	0.018	0.018333	0.000333	2.460850112	97.53914989	47.48 ± 0.54
	250	0.02	0.017	0.022	0.019667	0.001453	2.639821029	97.36017897	
	125	0.025	0.021	0.022	0.022667	0.001202	3.042505593	96.95749441	
	62.5	0.184	0.149	0.155	0.162667	0.010806	21.8344519	78.1655481	
	31.25	0.64	0.661	0.652	0.651	0.006083	87.38255034	12.61744966	
	15.62	0.749	0.738	0.743	0.743333	0.00318	99.77628635	0.223713647	
