# Supplementary material for: Investigating the in vitro antibacterial, antibiofilm, antioxidant, anticancer and antiviral activities of zinc oxide nanoparticles biofabricated from Cassia javanica
Source: PLoS One. 2024 Oct 1;19(10):e0310927. doi: 10.1371/journal.pone.0310927 (PMC11444386; doi:10.1371/journal.pone.0310927)
Supplement: S7 Table — (PDF) [file pone.0310927.s007.pdf]

S7 Table: Antiviral activity of Zinc oxide versus HSV1 and COX B4 viruses.

Test	ug/ml	O.D			Mean O.D	±SE	Viability %	Toxicity %	Viral activity %	Ant iviral effect %
Vero	-----	0.821	0.842	0.831	0.831333333	0.010503968	100	0	---	---
HSV1	-----	0.325	0.331	0.336	0.330666667	0.005507571	40.43	59.57	100	0
	62.5	0.653	0.663	0.662	0.659333333	0.005507571	79.66305656	20.33694344	34.13957267	65.86042733
	31.25	0.57	0.569	0.563	0.567333333	0.003785939	67.74969916	32.25030084	54.13849394	45.86150606
ZnO NPs	15.62	0.502	0.501	0.496	0.499666667	0.00321455	59.68712395	40.31287605	67.67311743	32.32688257
	7.81	0.422	0.426	0.435	0.427666667	0.006658328	52.3465704	47.6534296	79.99568508	20.00431492
	3.9	0.386	0.392	0.388	0.388666667	0.00305505	46.69073406	53.30926594	89.49012245	10.50987755

Test	ug/ml	O.D			Mean O.D	±SE	Viability %	Toxicity %	Viral activity %	Ant iviral effect %
Vero	-----	0.723	0.711	0.714	0.716	0.006244998	100	0	---	---
CoxB4	-----	0.322	0.334	0.329	0.328333333	0.006027714	45.81	54.19	100	0
	62.5	0.623	0.613	0.619	0.618333333	0.005033223	86.69467787	13.30532213	24.5530949	75.4469051
	31.25	0.537	0.531	0.522	0.53	0.007549834	73.1092437	26.8907563	49.62309707	50.37690293
ZnO NPs	15.62	0.417	0.421	0.431	0.423	0.007211103	60.36414566	39.63585434	73.14237745	26.85762255
	7.81	0.385	0.389	0.396	0.39	0.005567764	55.46218487	44.53781513	82.18825452	17.81174548
	3.9	0.356	0.351	0.349	0.352	0.003605551	48.87955182	51.12044818	94.33557516	5.66442484
